# Encapsulation of Hydrophobic Drugs in Shell-by-Shell Coated Nanoparticles for Radio- and Chemotherapy—An In Vitro Study

**DOI:** 10.3390/bioengineering7040126

**Published:** 2020-10-12

**Authors:** Stefanie Klein, Tobias Luchs, Andreas Leng, Luitpold V. R. Distel, Winfried Neuhuber, Andreas Hirsch

**Affiliations:** 1 Department of Chemistry and Pharmacy, Physical Chemistry I and ICMM, Friedrich-Alexander University of Erlangen Nuremberg, Egerlandstr. 3, D-91058 Erlangen, Germany; 2 Department of Chemistry and Pharmacy, Chair of Organic Chemistry II, Friedrich-Alexander University of Erlangen Nuremberg, Nikolaus-Fiebiger-Str. 10, D-91058 Erlangen, Germany; tobias.luchs@fau.de (T.L.); andreas.leng@fau.de (A.L.); andreas.hirsch@fau.de (A.H.); 3 Department of Radiation Oncology, Friedrich-Alexander University of Erlangen Nuremberg, Universitätsstr. 27, D-91054 Erlangen, Germany; luitpold.distel@uk-erlangen.de; 4 Institute of Anatomy and Cell Biology, Chair of Anatomy I, Friedrich-Alexander University of Erlangen Nuremberg, Krankenhausstr. 9, D-91054 Erlangen, Germany; winfried.neuhuber@fau.de

**Keywords:** shell-by-shell nanoparticles, TiO_2_ nanoparticles, Al_2_O_3_ nanoparticles, quercetin, drug delivery, radiotherapy, chemotherapy

## Abstract

Our research objective was to develop novel drug delivery vehicles consisting of TiO_2_ and Al_2_O_3_ nanoparticles encapsulated by a bilayer shell that allows the reversible embedment of hydrophobic drugs. The first shell is formed by covalent binding of hydrophobic phosphonic acid at the metal oxide surface. The second shell composed of amphiphilic sodium dodecylbenzenesulfonate emerges by self-aggregation driven by hydrophobic interactions between the dodecylbenzene moiety and the hydrophobic first shell. The resulting double layer provides hydrophobic pockets suited for the intake of hydrophobic drugs. The nanoparticles were loaded with the anticancer drugs quercetin and 7-amino-4-methylcoumarin. Irradiation with X-rays was observed to release the potential anticancer drugs into the cytoplasm. In Michigan Cancer Foundation (MCF)-10 A cells, quercetin and 7-amino-4-methylcoumarin acted as antioxidants by protecting the non-tumorigenic cells from harmful radiation effects. In contrast, these agents increased the reactive oxygen species (ROS) formation in cancerous MCF-7 cells. Quercetin and 7-amino-4-methylcoumarin were shown to induce apoptosis via the mitochondrial pathway in cancer cells by determining an increase in TUNEL-positive cells and a decrease in mitochondrial membrane potential after irradiation. After X-ray irradiation, the survival fraction of MCF-7 cells with drug-loaded nanoparticles considerably decreased, which demonstrates the excellent performance of the double-layer stabilized nanoparticles as drug delivery vehicles.

## 1. Introduction

After administration, conventional chemotherapeutic drugs are distributed throughout the whole body, which limits the dose that reaches the targeted tumor. Non-tumorigenic cells are also affected by these drugs, which results in cytotoxicity for healthy tissues and a poor tumor treatment [1,2]. One possibility to overcome this obstacle is to use nanoparticles (NPs) as a drug delivery system. Suited drug nanocarriers should remove limitations given by nonspecific drug distribution and targeting, lack of water solubility, low therapeutic indices, cytotoxicity, and multi drug resistance [1,3,4]. NPs with optimum sizes selectively accumulate in the tumor interstitium, while too small NPs are eliminated by the kidney and too large NPs are trapped in the reticuloendothelial system [1,5]. In comparison with healthy tissues, the higher probability of NPs being concentrated in the tumor tissues is due to permeable tumor vasculature and reduced lymphatic drainage, also known as the enhanced permeability and retention (EPR) effect [5,6,7]. Afterwards, most of the NPs enter the cancer cells via endocytosis by simultaneously impeding the recognition of the multi drug resistance transporter P-glycoprotein [1,8,9].

In vitro and in vivo studies have shown that suitably coated TiO_2_ NPs cause weak or no toxicity at low doses [2,10,11,12,13]. Simultaneously, they are able to kill bacteria [10], fungi [14], viruses [15], and cancer cells [16]. Anticancer drugs such as doxorubicin [17] and daunorubicin [18] could be directly attached to the TiO_2_ NP surface by forming complexes with the titanium ion. The drugs could also be transported in pores of mesoporous TiO_2_ NPs like daunorubicin in porous TiO_2_ nanowhiskers [3]. G. Devan and Venkatasubbu et al. [2] synthesized TiO_2_ NP coated with PEG and functionalized their surfaces with folic acid, which were afterwards loaded with paclitaxel. These TiO_2_ NPs show a good drug loading capacity but lack the possibility of a controlled release of the drugs at the tumor tissue by an internal or external stimulus.

In this contribution, we developed novel drug delivery nanocarriers consisting of TiO_2_ or Al_2_O_3_ NPs that were encapsulated by a smart bilayer shell using a shell-by-shell (SbS) coating procedure [19,20,21,22]. The bilayer shell was generated by covalently binding a hydrophobic n-alkyl phosphonic acid at the nanoparticle surface and subsequent self-aggregation of an amphiphilic building block. The hydrophilic surface structures of the second shell provide water dispersibility, whereas the hydrophobic chains of the phosphonic acid and the amphiphile form a hydrophobic pocket, which can embed hydrophobic drugs [23]. Our research goal was to validate the embedment of hydrophobic drugs within these pockets and to prove their controlled release in tumor cells.

The SbS-coated TiO_2_ and Al_2_O_3_ NPs were loaded with the hydrophobic drugs quercetin and 7-amino-4-methylcoumarin. Both compounds belong to the flavonoid group of secondary plant metabolites and possess, among others, anti-proliferative, anti-oxidant, and anti-tumor activities [24,25,26,27,28]. Coumarin derivatives suppress the cell proliferation by arresting the cell cycle in the G0/G1 [29,30] or G2/M [26] phase and quercetin by arresting the cell cycle in the G0/G1 [25,27], S [25] or G2/M [25,26,27] phase depending on the type of cancer cells. Additionally, coumarin derivatives and quercetin decrease the mitochondrial membrane potential [25,30] and therefore induce apoptosis via the mitochondrial pathway [25,30]. Both compounds are also known for their anti-oxidant activity by chelating transition metal ions and scavenging reactive oxygen species (ROS) [25,26,27,28,31]. Depending on their concentration and exposure time, quercetin or coumarin can also produce cytotoxic level of hydrogen peroxide in cancer cells and act as pro-oxidant [25,27,30,31,32].

The generation of ROS, the interaction with mitochondrial membrane and the potential to arrest the cells in the G2/M-phase of the cell cycle, which is the most radiosensitive cell cycle phase [33], make quercetin and coumarin good candidates for application as adjuvant anticancer in radiotherapy. C. Mirjolet et al. [34] reported that the fraction of cells in the G2/M phase increases after 24 h of exposure to titanate nanotubes. After irradiation with X-rays, the ROS generation inside cells rises in the presence of TiO_2_ NPs having anatase structure [35,36] generating mainly OH**^.^**, H**^.^**, and HO_2_ radicals [34,37]. Spherical TiO_2_ NPs can be used as a radio-sensitizer [16].

In this contribution, SbS-coated TiO_2_ and Al_2_O_3_ NPs were successfully loaded with the anticancer drugs quercetin (**G1**) and 7-amino-4-methylcoumarin (**G2**). The uptake of these nanocarriers by MCF-7 cells were confirmed by TEM and fluorescence microscopy. We could show that both kinds of nanocarriers, being SbS-coated TiO_2_ and Al_2_O_3_ NPs, are biocompatible. As an external stimulus, X-rays with a single dose of 1 Gy was used to release the anticancer drugs into the cytoplasm. In the non-tumorigenic MCF-10 A cells, quercetin and coumarin acted as a radioprotector and therewith improved the survival of the healthy cells. In contrast, low-dose X-ray irradiation drastically decreased the survival of the cancerous MCF-7 cells when containing the drug-loaded nanocarriers.

## 2. Materials and Methods

### 2.1. Chemicals

All chemicals and solvents were purchased from Sigma-Aldrich (St. Louis, Mo, USA), VWR (Darmstadt, Germany), Acros Organics (Geel, Belgium), Roth (Karlsruhe, Germany), and ABCR (Karlsruhe, Germany) and, if not otherwise noted, used without further treatment. Column chromatography was carried out on silica gel 60 (particle size, 0.04–0.063 mm) purchased from Macherey-Nagel (Düren, Germany). The titanium dioxide nanoparticles (anatase) were purchased from Nanograde^®^ (Stäfa, Switzerland) as a 20 wt.% dispersion in isopropanol. The aluminum oxide nanoparticles were purchased from Sigma-Aldrich (St. Louis, Mo, USA) as a 20 wt.% dispersion in isopropanol. The perylenediimide (**G3**) was prepared according to a literature procedure [38], (synthetic pathway Figure S1). Cell culture media, supplements and MitoSOX^TM^ Red Mitochondrial Superoxide Indicator were ordered from Thermo Fischer Scientific (Waltham, MA, USA). The In Situ Cell Death Detection Kit, TMR red were obtained from Roche Applied Science (Penzberg, Germany) and 5,5′,6,6′-Tetrachloro-1,1′,3,3′-tetraethyl-benzimidazolylcarbocyanine iodide (JC-1) from Biotium (Hayward, CA, USA).

### 2.2. Instruments

For dynamic light scattering (DLS) measurements and the determination of the zeta potential, a Zetasizer Nano series ZEN3600 (Malvern Instruments, Malvern, UK) with a 633 nm He-Ne laser was used. A Shimadzu UV-3102 PC was used for UV/Vis measurements. The TEM cell images were recorded on a LEO 906 TEM (Carl Zeiss AG, Oberkochen, Germany) and the fluorescence images on a Carl Zeiss Observer A.1 (BioSurplus, Inc., San Diego, CA, USA) with AxioCam and CoolLED pE-300. The diverse biological assays were performed by using a microplate reader (Synergy HT, BioTek Inc., Winuski, VT, USA). The cells were irradiated by an X-ray tube with a tungsten anode, which operates at an on-average energy of 34 keV (Comet MXR 160/0.4-3.0, DETEK, Inc., Clinton, MD, USA).

### 2.3. Surface Functionalization of Nanoparticles

#### 2.3.1. Experimental Procedure for the Preparation of Shell-by-Shell (SbS) Coated Nanoparticle Hybrids

According to a procedure previous reported by our group [19,39], the TiO_2_ or Al_2_O_3_ nanoparticles were exposed to a 5 mM solution of anchor molecule PAC_16_ in isopropanol. In a standard experiment, 7.5 mL of a 0.25 wt.% dispersion of TiO_2_ or Al_2_O_3_ in isopropanol was treated with 5 mL of a 5 mM solution of PAC_16_ in isopropanol. This mixture was subjected to sonication at 21 °C for 30 min followed by centrifugation (14,000 rpm, 10 min) and an additional washing step. The washing step included redispersion in isopropanol (0.15 wt.%), sonication for 5–10 min and centrifugation (14,000 rpm, 10 min). For further characterization the particles were dried at 75 °C overnight or redispersed in the solvent of choice for DLS and zeta potential measurements or further synthetic procedures. When transferred into different solvents, the particle concentration was kept constant at approximately 0.15 wt.%. The as synthesized core-shell nanoparticles [TiO_2_-PAC_16_] or [Al_2_O_3_-PAC_16_] were dispersed in water (0.15 wt.%) and exposed to amphiphile shell**1** (Sodium dodecylbenzene sulfonate) in 1 mM concentration. The overall volume was 10 mL, corresponding to 15 mg nanoparticle on average. In a routine experiment, the amphiphile was dissolved in 5 mL water and added to 5 mL of a 0.3 wt.% nanoparticle dispersion. This procedure included sonication for 30 min at 21 °C, followed by centrifugation (14,000 rpm, 10 min) to remove free amphiphile and a further washing step. The final nanoparticle hybrids were dispersed in aqueous media for DLS and zeta analysis or further synthetic procedures.

#### 2.3.2. Incorporation of Hydrophobic Molecules

An aqueous 0.15 wt.% dispersion (10 mL) of the SbS coated nanoparticles TiO_2_-PAC@shell**1** or Al_2_O_3_-PAC_16_@shell**1** was exposed to **G1**, **G2**, or **G3** dissolved in acetone (500 µL). The dispersion was stirred at room temperature in an open vial for 24 h to facilitate slow evaporation of the volatile components. The respective excess hydrophobic drug precipitated in the solution and was removed by filtration. The filtrate was centrifuged (14,000 rpm, 10 min) and redispersed in water until no free guest was observed in the supernatant. For analysis by UV/Vis, DLS, and zeta potential measurements or further processing, the particles were dispersed in 10 mL of water at a particle concentration of approximately 0.15 wt.%. For the quantification of incorporated guest molecule, 1 mL of an aqueous 0.15 wt.% dispersion of loaded nanocarrier was sedimented by centrifugation. The residue was treated with 1 mL of a suitable organic solvent and sonicated for 10 min (acetone for NP loaded with **G1**, acetonitrile for NP loaded with **G2**, and toluene for NP loaded with **G3**). The NPs were removed by centrifugation and the extraction process was repeated one more time. After dilution, the incorporated guest amount was quantified via UV/Vis spectroscopy. The remaining nanoparticles turned colorless after the organic extraction steps, indicating a complete removal of the guest molecules. As further control experiment, we conducted a third organic extraction step and did not observe any free guest in the organic phase. For the drug release experiment, 7 mL of an aqueous 0.15 wt.% dispersion of [TiO_2_-PAC_16_]@guest **G3**@shell**1** were centrifuged and then redispersed in 2 mL water. The aqueous dispersion was overlaid with 10 mL toluene and 250 μL acetone were added. The mixture was stirred at room temperature. Aliquots of the organic phase were taken in several time intervals and then diluted with toluene for UV/Vis measurements.

### 2.4. Cell Culture

The culture conditions of the human breast cancer MCF-7 cells and MCF-10 A cells were described by S. Klein at al [40,41]. The MCF-7 cell line was provided by Dr. Erasmus Schneider, Wadsworth Center New York State Department of Health and the MCF-10 A cells were donated by Dr. Simone Brabletz, Nicolaus-Fiebiger-Center for Molecular Medicine, University Erlangen-Nuremberg.

### 2.5. Cell Preparation for Microscope Images

#### 2.5.1. TEM Images

The cells were grown in cell culture flask to 90% confluency. The medium was replaced by one containing 10 µg/mL [TiO_2_-PAC_16_]@guest **G1**@shell**1** (0.71 μg/mL quercetin). After 24 h, the cells were washed thrice with ice cold phosphate buffered saline (PBS) containing 1mM ethylene diamine tetraacetic acid (EDTA). The cells were detached with Trypsin/EDTA solution, fixed with 2.5% glutaraldehyde in PBS at 4 °C for 8 h, and post-fixed in 1% osmium tetroxide and 3% potassium ferricyanide at room temperature. Afterwards, the cells were dehydrated in alcohol and embedded in Epon. Sections of 60–70 nm were cut using an ultramicrotome. Non-contrasted silver-grey ultrathin sections were imaged.

#### 2.5.2. Fluorescent Microscope

MCF-7 and MCF-10 A cells were grown on 4-well chamber slides (10,000 cells/well) and kept in the incubator for 24 h for attachment. An amount of 10 μg/mL [Al_2_O_3_-PAC_16_]@guest **G3**@shell**1** (0.57 μg/mL perylenediimide) or [TiO_2_-PAC_16_]@guest **G3**@shell**1** (0.31 μg/mL perylenediimide) was added. The cells were incubated for 8 h. The cells were fixed with 1:1 mixture of 4% paraformaldehyde/PBS and medium for 10 min and only 4% paraformaldehyde/PBS for 10 min. The cells were washed with PBS, permeabilized with 1% Triton-X. The cell nucleus was stained with Hoechst 33258 (1 µg/mL in PBS).

### 2.6. MTT Assay

MCF-7 and MCF-10 A cells were seeded into 96-well plates at a density of 20,000 cells/well and put in the incubator for 24 h. Afterwards, the cells were treated with 25 μg/mL of [TiO_2_-PAC_16_]@shell**1**, [TiO_2_-PAC_16_]@guest **G1**@shell**1** (1.78 μg/mL quercetin), [TiO_2_-PAC_16_]@guest **G2**@shell**1**, [Al_2_O_3_-PAC_16_]@shell**1** or [Al_2_O_3_-PAC_16_]@guest **G1**@shell**1** (1.15 μg/mL quercetin) for 24 h. 50 μL/well of the yellow thiazolyl blue tetrazolium bromide solution (0.5 mg/mL in PBS) was added. After 2 h the solution was carefully removed. The blue formazan crystals were dissolved by addition of 100 μL dimethyl sulfoxide (DMSO). The absorbance was measured at 590 nm.

### 2.7. Radical Detection Assays

MCF-7 and MCF-10 A cells were cultivated in 96-well plates (20,000 cells/well). After 24 h, 25 μg/mL of [TiO_2_-PAC_16_]@shell**1**, [TiO_2_-PAC_16_]@guest **G1**@shell**1** (1.78 μg/mL quercetin), [TiO_2_-PAC_16_]@guest **G2**@shell**1**, [Al_2_O_3_-PAC_16_]@shell**1**, or [Al_2_O_3_-PAC_16_]@guest **G1**@shell**1** (1.15 μg/mL quercetin) were added and incubated for another 24 h. The medium was removed and the cells were treated with the respective fluorescent dye (see their sections). Half of the plate was covered with a lead plate and the other half was irradiated with a single dose of 1 Gy. The results were normalized by Hoechst 33528 nuclear staining.

#### 2.7.1. Superoxide

The generation of superoxide radical (O_2_^•−^) was measured using the MitoSOX^TM^ Red mitochondrial superoxide indicator and following the manufacture’s protocol. In short, the dye was dissolved in DMSO to a final concentration of 2.5 mM and diluted to 5 µM in Hank’s balanced salt solution (HBSS). The cells were incubated in this solution for 10 min. After X-ray irradiation, they were washed and 100 µL of HBSS was added. The fluorescence intensity of the MitoSOX^TM^ Red mitochondrial superoxide indicator was measured upon excitation at 530 nm and detection at 590 nm.

#### 2.7.2. ROS

The 2′,7′-dichlorofluorescein diacetate (DCFH-DA) was dissolved in DMSO to obtain a 0.01 M stock solution. This solution was diluted to 100 μM with DMEM (Dulbecco’s Modified Eagle Medium with 4.5 g/L D-Glucose, L-glutamine and 25 mM (4-(-2-hydroxyethyl)-1-piperazineethanesulfonic acid (HEPES) without any additives. The cells were loaded with the DCFH-DA solution for 30 min. Upon entering the cells, the diacetate groups were cleaved and the DCFH dye was trapped in the cells. Afterwards the wells were washed with PBS and 100 μL PBS was added per well. Intracellular DCFH was oxidized by ROS to the fluorescent DCF dye. After the irradiation with a single dose of 1 Gy, the DCF dye was excited at 480 nm, and its fluorescence emission was detected at 528 nm.

### 2.8. Mitochondrial Depolarization

The impact of the SBS nanoparticle hybrids on the mitochondrial membrane potential was measured with the fluorescent probe JC-1. MCF-7 and MCF-10 A cells were seeded in 96-well plates, treated with the nanocarriers and irradiated with a single dose of 1 Gy as described before. Afterwards the cells were incubated at 37 °C for 30 min with JC-1 (1 μg/mL in cell culture medium. The cells were washed twice with Krebs-Ringer buffer and finally 100 μL Krebs-Ringer buffer was added per well. The fluorescence intensity of the green JC-1 monomer (485 nm/528 nm) and that of the red JC-1 aggregates (530 nm/590 nm) was detected. The ratio aggregate to monomer determines the degree of mitochondrial depolarization.

### 2.9. TUNEL Assay

The MCF-7 and MCF-10 A cells were kept in 96-well plates and incubated overnight. Afterwards, the medium was replaced with one containing 25 μg/mL of [TiO_2_-PAC_16_]@shell**1**, [TiO_2_-PAC_16_]@guest **G1**@shell**1** (1.78 μg/mL quercetin), [TiO_2_-PAC_16_]@guest **G2**@shell**1**, [Al_2_O_3_-PAC_16_]@shell**1** or [Al_2_O_3_-PAC_16_]@guest **G1**@shell**1** (1.15 μg/mL quercetin). The TUNEL assay was performed according to the manufacturer’s instructions. Afterwards, the cell nuclei of all cells were stained with Hoechst 33258 (1 μg/mL in PBS) to determine the ratio of apoptotic cells.

### 2.10. Clonogenic Cell Survival Assay

This experiment was based on a method described in detail by N.A.P. Franken et al. [42]. Both cell lines were grown in 6-well plates and incubated overnight 25 μg/mL of [TiO_2_-PAC_16_]@shell**1**, [TiO_2_-PAC_16_]@guest **G1**@shell**1** (1.78 μg/mL quercetin), [TiO_2_-PAC_16_]@guest **G2**@shell**1**, [Al_2_O_3_-PAC_16_]@shell**1** or [Al_2_O_3_-PAC_16_]@guest **G1**@shell**1** (1.15 μg/mL quercetin). After X-ray irradiation between 0 and 3 Gy, the cells were detached, seeded, and grown in 6-well plates for 2 weeks to form colonies. The colonies were fixed and stained with a mixture of 0.5% (*w*/*v*) crystal violet in 50/50 methanol/water for 30 min. The count of colonies containing >50 cells was used for the calculation of the surviving fraction (SF). The survival curves were fitted to a linear quadratic function (ln SF = −(αD + βD^2^)). To quantify the X-ray enhancing effect the dose modifying factor (DMF) was calculated from the X-radiation survival curves by taking the ratio of radiation doses at the 50% survival level (NP-treated radiation dose divided by the control radiation dose). DMF values <1 indicate an X-ray enhancing effect.

## 3. Results and Discussion

### 3.1. Characterization of the Shell-by-Shell Coated Nanocarriers

**Figure 1:**
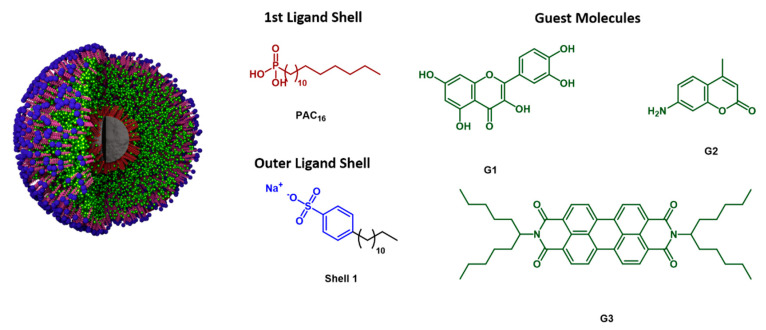
Shell-by-Shell (SbS) coated nanocarrier assemblies that are loaded with guest molecules **G1** (quercetin), **G2** (7-amino-4-methylcoumarin), and **G3** (perylendiimide). Inorganic nanoparticle core: TiO_2_ or Al_2_O_3._

TiO_2_ NPs with a specific surface area of 46 m^2^/g (Table S1) and Al_2_O_3_ NPs with a specific surface area of 100.5 m^2^/g (Figure S2) were selected for our studies. Following a previously reported procedure, these NPs were covalently functionalized with hexadecylphosphonic acid (PAC_16_) in a wet chemistry approach [39]. After multiple washing steps consisting of redispersion in isopropanol and centrifugation, the core-shell nanoparticles [TiO_2_-PAC_16_] or [Al_2_O_3_-PAC_16_] were treated with an aqueous 1 mM solution of sodium dodecylsulfonate (shell**1**) to yield the nanoparticle hybrids [TiO_2_-PAC_16_]@shell**1** or [Al_2_O_3_-PAC_16_]@shell**1** [19]. The unique architecture resulting from this shell-by-shell (SbS) coating procedure enables the incorporation of hydrophobic guest molecules into the shell of these nanoparticles (Figure 1). This was previously demonstrated for a series of simple apolar compounds by Luchs et al. [20]. Our objective was to verify and validate the reversible incorporation of hydrophobic drugs within the SBS coating of TiO_2_ and Al_2_O_3_ nanoparticles, where the potentially successful drug delivery performance comprises the controlled drug transport and release into cancer cells. In addition to the anticancer drugs quercetin and 7-amino-4-methylcoumarin, a fluorescent perylendiimide was used to visualize the drug delivery and release performance of SbS coated nanoparticles by means of fluorescence microscopy and spectroscopy. To facilitate the incorporation, the hydrophobic guest molecules **G1** (quercetin), **G2** (7-amino-4-methylcoumarin) and **G3** (perylendiimide) were dissolved in a minimal amount of acetone and added to an aqueous dispersion of [TiO_2_-PAC_16_]@shell**1** or [Al_2_O_3_-PAC_16_]@shell**1**. The mixtures were sonicated and stirred in an open vial to enable slow evaporation of acetone. The non-complexed hydrophobic guest molecules precipitated out of solution and were removed by filtration. Further washing steps were conducted until no free guest was observed in the supernatant. Generally, the incorporation of hydrophobic guest molecules into the shells of [TiO_2_-PAC_16_]@shell**1** or [Al_2_O_3_-PAC_16_]@shell**1** led to an increase in hydrodynamic diameter and a color change from white to yellow (**G1**), light blue (**G2**), or red (**G3**) (Figure S3).

**Figure 2:**
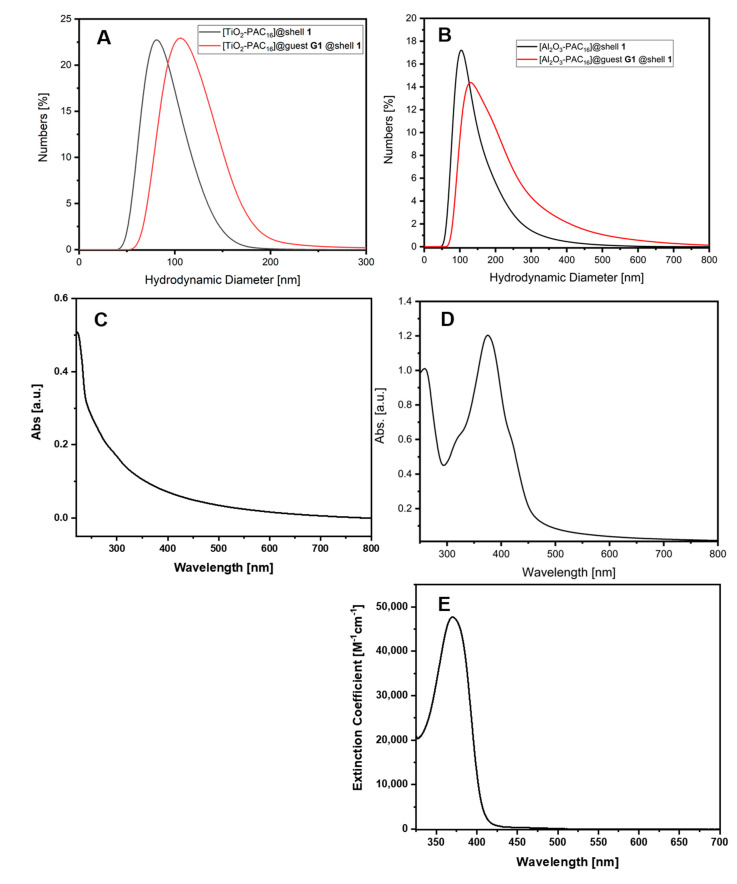
Comparison of the hydrodynamic diameter of the unloaded nanocarriers and the nanocarriers loaded with **G1** (quercetin): (**A**) TiO_2_ nanocarriers, (**B**) Al_2_O_3_ nanocarriers; (**C**) UV/Vis spectrum of the unloaded nanocarrier [Al_2_O_3_-PAC_16_]@shell**1**; (**D**) loaded nanocarrier [Al_2_O_3_-PAC_16_]@guest **G1**@shell**1** and quercetin in acetone (**E**).

The loaded nanocarrier [TiO_2_-PAC_16_]@guest **G1**@shell**1** displayed a hydrodynamic diameter of 106 nm while the empty SbS coated NPs [TiO_2_-PAC_16_]@shell**1** previously displayed a hydrodynamic diameter of 81 nm in water (Figure 2A). Despite the strong yellow color, [TiO_2_-PAC_16_]@guest **G1**@shell**1** yielded the same optical absorption data as the white dispersion of [TiO_2_-PAC_16_]@shell**1** which we attribute to light scattering effects (Figure S4). Similar observations were made for [TiO_2_-PAC_16_]@guest **G2**@shell**1** (Figures S4 and S5). In contrast to the TiO_2_-based nanocarriers, the optical absorption spectrum of [Al_2_O_3_-PAC_16_]@guest **G1**@shell**1** displays strong contributions of **G1** with a maximum at 375 nm (Figure 2C–E). This is also accompanied by an increase in hydrodynamic diameter from 104 nm to 140 nm (Figure 2B). Compared to [TiO_2_-PAC_16_]@guest **G1**@shell**1**, the loaded nanocarrier [Al_2_O_3_-PAC_16_]@guest **G1**@shell**1** was less stable in aqueous dispersion. To prove the stability of the nanocarrier dispersions in biological fluids, DLS measurements were also conducted in medium supplemented with 10% fetal serum as used in the biological experiments. Since proteins and other biological macromolecules build an additional shell around the nanocarrier systems, the so called protein corona, the hydrodynamic diameter of the nanocarriers systems in medium increased (Table 1, Tables S2 and S3, Figures S6 and S7). The loading capacity of [TiO_2_-PAC_16_]@guest **G1**@shell**1** and [Al_2_O_3_-PAC_16_]@guest **G1**@shell**1** could be determined by centrifugation of the loaded nanocarrier dispersions followed by extraction of the sediment with acetone. After dilution, the exact amount of incorporated guest molecules could be derived from UV/Vis spectroscopy (Table 1). The loading capacity of [TiO_2_-PAC_16_]@guest **G1**@shell**1** with 7.1% is higher than that of the aluminum oxide counterpart.

**Figure 3:**
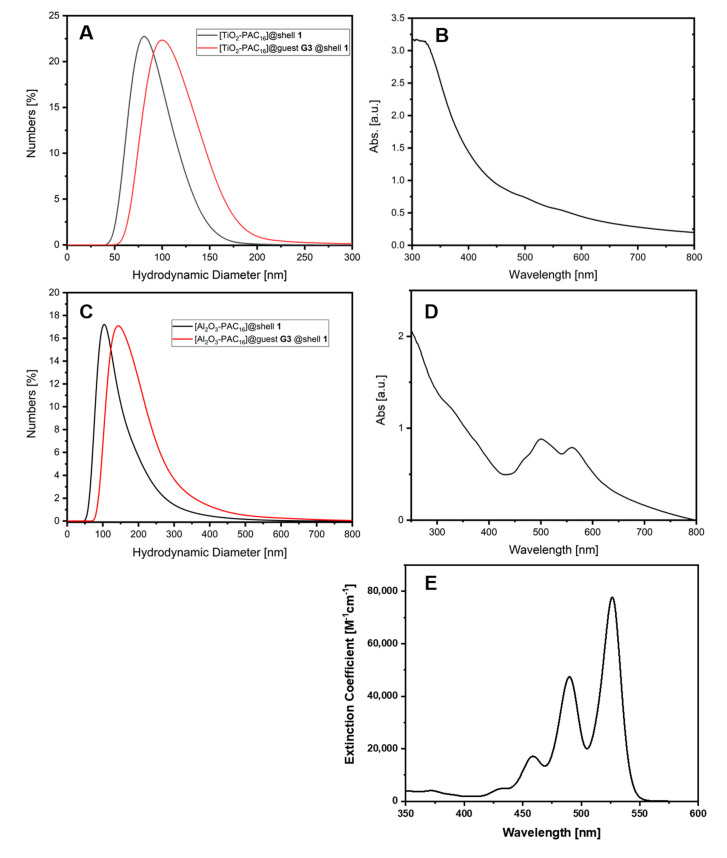
Comparison of the hydrodynamic diameters of the unloaded nanocarriers with that of the nanocarriers loaded with **G3** (perylendiimide): (**A**) TiO_2_ nanocarriers, (**C**) Al_2_O_3_ nanocarriers; UV/Vis spectrum of the loaded nanocarriers [TiO_2_-PAC_16_]@guest **G3** @shell**1** (**B**) or [Al_2_O_3_-PAC_16_]@guest **G3**@shell**1** (**D**) and the perylendiimide (**G3**) in toluene (**E**).

**Figure 4:**
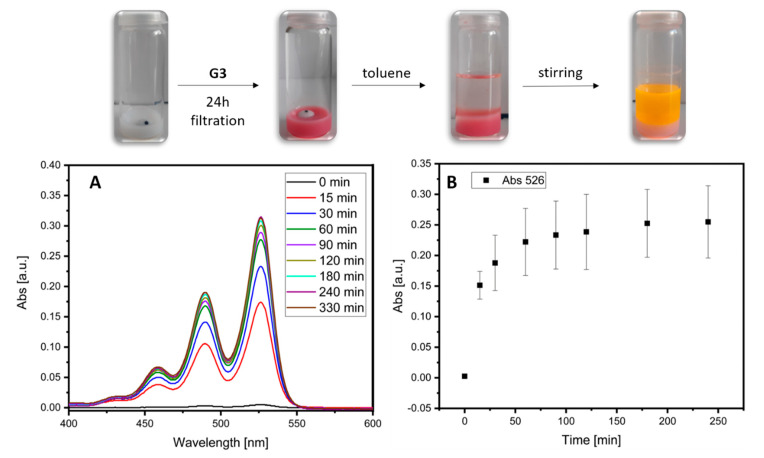
Time-dependent release of **G3** from the loaded nanocarrier [TiO_2_-PAC_16_]@guest **G3** @shell**1** in a biphasic system (water/toluene). (**A**) Absorption spectra taken from diluted aliquots of the organic phase during measurement series 1. (**B**) Mean absorption at 526 nm plotted vs time.

Incorporation of the hydrophobic PDI **G3** into the shell of the empty nanocarrier [TiO_2_-PAC_16_]@shell**1** resulted in a color change of the previously white aqueous nanoparticle dispersion. The excess of guest molecules precipitated out and was removed by filtration. After further washing steps consisting of dispersion in water followed by centrifugation, a stable aqueous dispersion with a red color was obtained, indicating the successful formation of [TiO_2_-PAC_16_]@guest **G3**@shell**1** (Figure S8). DLS measurements clearly showed an increase in the hydrodynamic diameter of the nanoparticle dispersion from 81 nm for the pristine nanocarrier to about 100 nm after incorporation of the guest molecules (Figure 3A). Despite the clearly observable red color of the dispersion, UV/Vis measurements of [TiO_2_-PAC_16_]@guest **G3**@shell**1** in water only exhibit minor features of the PDI **G3** in the range of 450 to 600 nm (Figure 3B). We attribute this observation to light scattering effects of the TiO_2_ NPs. The loading capacity of [TiO_2_-PAC_16_]@guest **G3**@shell**1** and [Al_2_O_3_-PAC_16_]@guest **G3**@shell**1** was determined by extraction with toluene (Table 1). The loading capacity of the Al_2_O_3_ NPs is higher than that of the TiO_2_ NPs-based ones in case of G3 incorporation. However, dispersions of [TiO_2_-PAC_16_]@guest **G3**@shell**1** are relatively stable while the Al_2_O_3_ nanocarriers with G3 show agglomeration. To demonstrate the solvent dependent reversibility of the hydrophobic drug incorporation in SbS-coated nanoparticles, an aqueous dispersion of [TiO_2_-PAC_16_]@guest **G3**@shell**1** was overlaid with toluene. Stirring of this biphasic system led to the release of encapsulated **G3** from the aqueous phase into the toluene phase which could be followed by UV/Vis spectroscopy. Initially the release of **G3** occurred rapidly but slowed down significantly after 30 min until an equilibrium was reached after 3 h (Figure 4). Along with previous studies, these findings suggest that the SbS-coated nanocarriers [TiO_2_-PAC_16_]@shell**1** exhibit an excellent performance for the transport and controllable release of hydrophobic molecules in in the cytoplasm of tumor cells. [23,43]. The loaded nanocarrier [Al_2_O_3_-PAC_16_]@guest **G3** @shell**1** was prepared through the same procedure as the TiO_2_-based nanocarriers. Similarly, the incorporation of **G3** resulted in a color change of the previously colorless dispersion of [Al_2_O_3_-PAC_16_]@shell**1** to a red dispersion of [Al_2_O_3_-PAC_16_]@guest **G3** @shell**1** in water. The hydrodynamic diameter increased from 104 nm to 142 nm upon incorporation of the hydrophobic guest (Figure 3C). In contrast to [TiO_2_-PAC_16_]@guest **G3**@shell**1**, the UV/Vis measurement of an aqueous dispersion of [Al_2_O_3_-PAC_16_]@guest **G3**@shell**1** clearly showed characteristic features of PDI (**G3**) in the range of 450–640 nm (Figure 3D).

### 3.2. Cellular Uptake of the Nanocarriers

**Figure 5:**
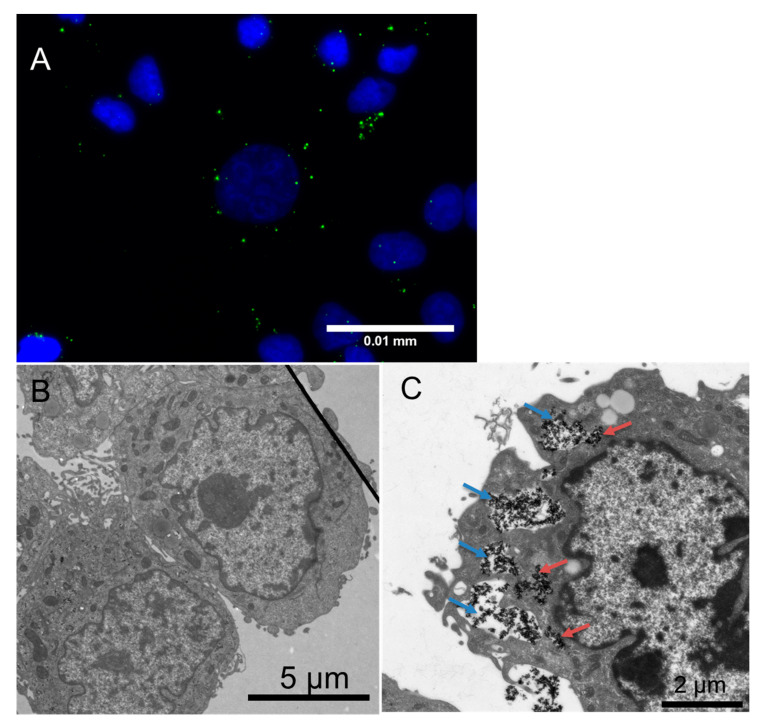
Fluorescence microscope image (**A**) of MCF-7 cells loaded with [Al_2_O_3_-PAC_16_]@guest **G3**@shell**1** (cell nucleus (blue) nanocarrier (green); TEM images of MCF-7 cells without NPs (**B**) with [TiO_2_-PAC_16_]@guest **G1**@shell**1** (**C**) in vesicles (blue arrows) and inside the cytoplasm (red arrows)

The cellular uptake of the nanocarriers was examined using fluorescence and transmission electron microscopy. Breast cancer cells (MCF-7) and non-tumorigenic breast epithelial cells (MCF-10 A) were incubated with the fluorescent [Al_2_O_3_-PAC_16_]@guest **G3**@shell**1** for 4 h. In the fluorescence microscope image (Figure 5A and Figure S9), the green spots of the [Al_2_O_3_-PAC_16_]@guest **G3**@shell**1** were found around the blue stained cell nucleus indicating a cellular uptake of the nanocarriers. To make sure that the nanocarriers are not only attached to the cell surface, TEM images of MCF-7 cells without NPs (Figure 5B) and loaded with [TiO_2_-PAC_16_]@guest **G1**@shell**1** were recorded (Figure 5C). In comparison to the MCF-7 cells without NPs (Figure 5B), the [TiO_2_-PAC_16_]@guest **G1**@shell**1** are visible as dark dots inside the MCF-7 cells (Figure 5C). The nanocarriers were taken up by the cells via endocytosis, which is the main cellular entry mechanism for NPs [44,45,46,47]. Endocytosis leads to an ingestion of the NPs from the extracellular space by an invagination of the plasma membrane and subsequent forming of intracellular vesicles [44,47]. Therefore, the cellular uptake via endocytosis is evident from the TEM image in Figure 5C illustrating the NPs inside vesicles (Figure 5C: blue arrows). The transcellular trafficking of endocytic vesicles to different compartments follows the endosomal network, which includes the homotypic fusions of primary endocytic vesicles to form various types of endosomes [44,47,48]. To avoid the transport back to the plasma membrane or the degradation in the lysosomes, NPs have to escape the endosomal network. In the TEM micrograph, the nanocarriers were also observed inside the cytoplasm (Figure 5C: red arrows). This indicates that the nanocarriers escaped from the endocytic pathway, which enabled them to deliver their hydrophobic drugs into the cytoplasm.

### 3.3. Biocompatibility before and after X-Ray Radiation

**Figure 6:**
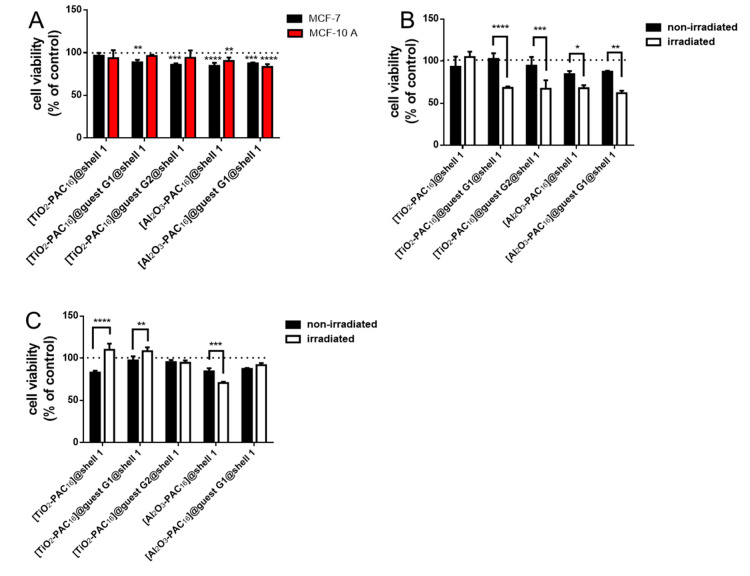
Biocompatibility of the nanocarriers for MCF-7 and MCF-10 A cells (**A**), cell viability in nanocarriers loaded MCF-7 (**B**) and MCF-10 A (**C**) cells before and after irradiation with a single dose of 1 Gy, n = 6, *: *p* < 0.05, **: *p* < 0.01, ***: *p* < 0.001, ****: *p* < 0.0001.

MCF-7 and MCF-10 A cells were treated with 25 μg/mL nanocarriers and the cell viability was tested after 24 h. The unloaded nanocarriers as well as those loaded with guest molecule **G1** and **G2** are biocompatible for both cell lines (Figure 6A). The [Al_2_O_3_-PAC_16_]@shell**1** were a little more toxic than the [TiO_2_-PAC_16_]@shell**1** for MCF-7 and MCF-10 A cells. The cell viability of cancerous MCF-7 cells when containing [TiO_2_-PAC_16_]@shell**1** (96%) differs scarcely from the cell viability of MCF-7 cells loaded either with [TiO_2_-PAC_16_]@guest **G1**@shell**1** (90%) or [TiO_2_-PAC_16_]@guest **G2**@shell**1** (88%). Since the anticancer drugs quercetin (**G1**) and 7-amino-4-methylcoumarin (**G2**), which were dissolved in DMSO and added to the cell medium, significantly lowered the cell viability of MCF-7 cells significant more (Figure S10), the hydrophobic drugs remained adsorbed inside the nanocarriers after 24 h. Obviously an external stimulus is needed to open the shell around the nanocarriers and thereupon to release the anticancer drugs. As an external stimulus, X-rays were used. Our former research activities on applying iron oxide NPs in radiotherapy was based on the phenomenon that X-rays ablate surface structures from the iron oxide NPs. This implies that X-ray irradiation activates surface Fe^2+^ and Fe^3+^ that catalyze the Fenton and Haber-Weiss reaction, and highly toxic ROS are generated [40,49,50]. Hence, we expected that X-radiation damages the bilayer shell system of the intracellular nanocarriers and the cancer drugs will be released to the cytoplasm. To prove these expectations, the MCF-7 and MCF-10 A cells were incubated with unloaded and loaded nanocarriers system for 24 h and half of the samples were subsequently irradiated with a single dose of 1 Gy and the other half were kept non-irradiated as negative control. The cell viability of both test groups was determined and the value of the cells in medium without nanocarriers was used as 100%, the value at 0 Gy for the non-irradiated group and the value at 1 Gy for the irradiated group. The cell viability of the MCF-7 cells with internalized nanocarrier [TiO_2_-PAC_16_]@shell**1** without any drugs remained the same whether they were irradiated or not (Figure 6B). This result demonstrated that unloaded [TiO_2_-PAC_16_]@shell**1** are biocompatible. However, the nanocarriers [TiO_2_-PAC_16_]@guest **G1**@shell**1** and [TiO_2_-PAC_16_]@guest **G2**@shell**1**, when incorporated in the cancerous MCF-7 cells, drastically decreased the survival of these cells. This indicated a release of the guest molecules **G1** and **G2** from the hydrophobic pocket after irradiation into the cytoplasm after irradiation of the cells, where they developed their anti-tumor effects. The difference between unloaded and loaded Al_2_O_3_ nanocarriers was not as visible as for the TiO_2_ nanocarriers, since the [Al_2_O_3_-PAC_16_]@shell**1** NPs significantly diminished the cell survival after irradiation. In contrast to the MCF-7 cells, the cell viability of the non-tumorigenic MCF-10 A cells was not affected by the unloaded and loaded TiO_2_ nanocarrier systems at 0 or 1 Gy (Figure 6C). Quercetin (**G1**) can act as pro-oxidant or antioxidant depending on its intracellular concentration [27,30,31,32]. In case of coumarin derivatives, not only the dose is responsible for the pro- or antioxidant effect but also the structure of the used coumarin derivative [51,52]. Therefore, the coumarin (**G2**) shows a similar concentration dependency as quercetin (**G1**). As an antioxidant, it protects the non-tumorigenic MCF-10 A cells from the harmful radical generation of the X-rays. The dose of the quercetin released into the MCF-10 A cells was in the range so that the cells were protected against X-radiation. This effect was even larger for the Al_2_O_3_ nanocarriers. While the unloaded [Al_2_O_3_-PAC_16_]@shell**1** damaged the MCF-10 A cells after irradiation, the cell viability even slightly increased after irradiation of MCF-10 A cells with [Al_2_O_3_-PAC_16_]@guest **G1**@shell**1**.

### 3.4. Changes of ROS and Superoxide Concentration

**Figure 7:**
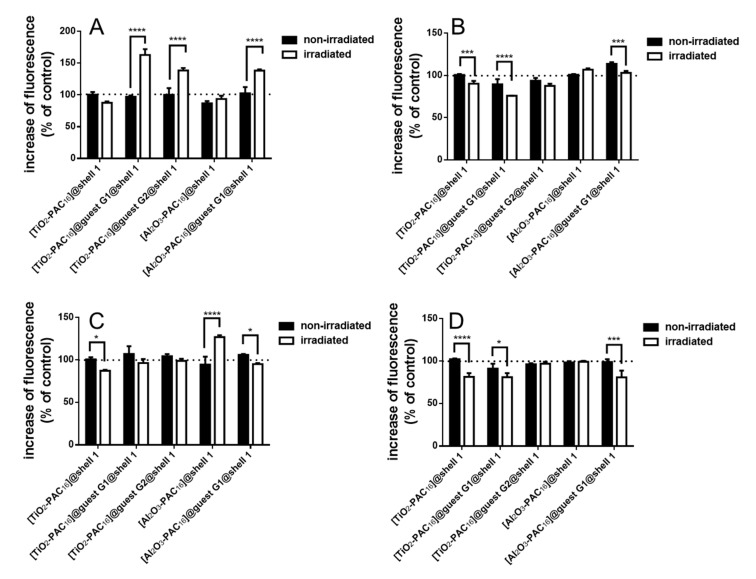
Changes of the ROS level in MCF-7 (**A**) and MCF-10 A cells (**C**) and the superoxide level in MCF-7 (**B**) and MCF-10 A (**D**) cells before and after irradiation with a single dose of 1 Gy, n = 6, *: *p* < 0.05, ***: *p* < 0.001, ****: *p* < 0.0001.

To clarify the anti- or pro-oxidative effect of the guest molecules, the change of the intracellular ROS levels was measured. The unloaded nanocarriers [TiO_2_-PAC_16_]@shell**1** slightly reduced the ROS concentration in both cell lines after X-ray irradiation (Figure 7A,C), whereas the unloaded [Al_2_O_3_-PAC_16_]@shell**1** nanocarriers increased the ROS concentration in the MCF-10 A cells (Figure 7C), when irradiated with 1 Gy. MCF-7 cells with [TiO_2_-PAC_16_]@guest **G1**@shell**1** and [TiO_2_-PAC_16_]@guest **G2**@shell**1** exhibit an increased ROS formation after irradiation in contrast to the MCF-10 A cells. This is in line with the results of the cell viability assay, where the cell survival of the MCF-7 is smaller than that of the MCF-10 A cells. Also, the concentration of the drugs released to the cytoplasm of MCF-7 cells is high enough for a pro-oxidative effect of **G1** and **G2**. The same behavior can be found for MCF-7 and MCF-10 A cells with [Al_2_O_3_-PAC_16_]@guest **G1**@shell**1**. Bioflavonoids, especially quercetin, are known for their ability to scavenge the superoxide anions [53,54]. Therefore, the change of the superoxide generation was measured after irradiation with a single dose of 1 Gy. X-ray irradiation induces the formation of superoxide by mitochondrial membrane depolarization. The superoxide level significantly decreased after irradiation of the MCF-7 and MCF-10 A cells loaded with [TiO_2_-PAC_16_]@guest **G1**@shell**1** or with [Al_2_O_3_-PAC_16_]@guest **G1**@shell**1** (Figure 7B,C). For irradiated cells with [TiO_2_-PAC_16_]@guest **G2**@shell**1**, only a slight decline in superoxide concentration was observed for MCF-7 cells, but not for MCF-10 A cells. According to these results quercetin turned out to perform as a superior superoxide scavenger in comparison with 7-amino-4-methylcoumarin. Superoxide was disproportionated by quercetin to hydrogen peroxide and O_2_. This explains the increase in ROS generation inside the MCF-7 cells (Figure 7A). In contrast to the MCF-10 A cells, the level and activity of hydrogen peroxide scavenging enzymes such as catalase or glutathione (GSH) peroxidase are reduced in MCF-7 cells. Therefore, cancer cells like MCF-7 possess per se higher intracellular hydrogen peroxide levels and cannot cope with additional formation of hydrogen peroxide.

### 3.5. Mitochondrial Membrane Potential and DNA Fragmentation

**Figure 8:**
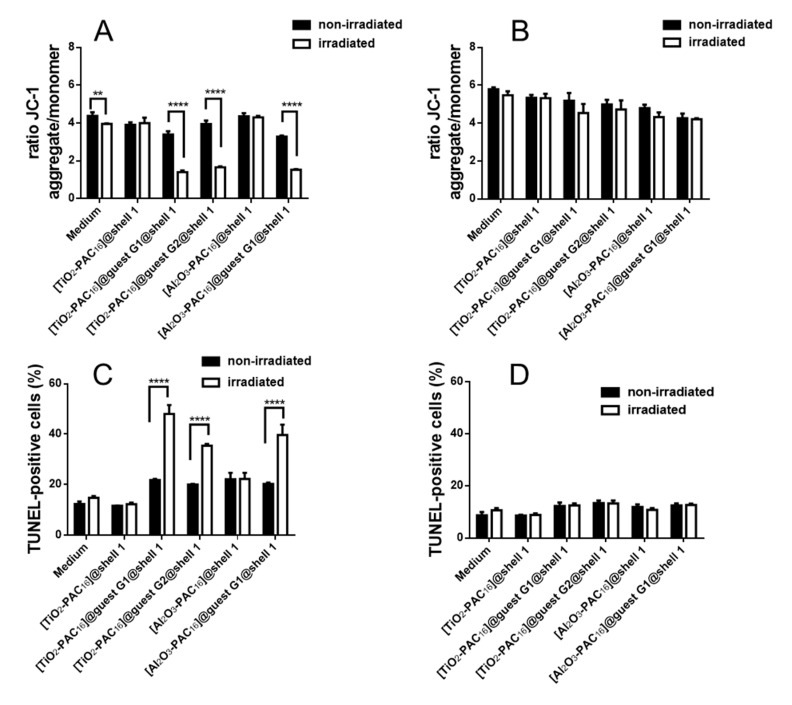
Changes of the mitochondrial membrane potential in MCF-7 (**A**) and MCF-10 A (**B**) cells expressed as ratio of fluorescence JC-1 aggregates (red) to monomers (green) and increase of DNA strand breaks in MCF-7 (**C**) and MCF-10 A (**D**) cells labeled by the TUNEL-assay, n = 6, **: *p* < 0.01, ****: *p* < 0.0001.

The increase of the ROS production caused by quercetin or coumarin is accompanied by a depolarization of the mitochondrial membrane potential [25,28,30]. Mitochondrial membrane potential (MMP) changes were measured with the dye JC-1 by accumulating inside the mitochondria. At high concentrations, this dye forms aggregates, which exhibit a red fluorescence. In case of damaged mitochondria, the membrane permeability is increased and the JC-1 dye is released from the mitochondria, leading to a much smaller concentration of this dye inside damaged mitochondria. For sufficiently lowered concentrations, JC-1 cannot aggregate and exists in its green fluorescent monomeric form. Thus, the ratio of red to green fluorescence determines the integrity of the mitochondrial membrane and, therewith, the change in its potential. [55]. X-ray radiation does not only induce ROS formation and DNA strand breaks, but also alters the functionalities of other cell organelles like the mitochondria. X-radiation increases mitochondrial ROS formation and membrane permeabilization [56]. MCF-7 cells cultivated in cell medium without any nanoparticles showed a significant decrease in the MMP (Figure 8A). No such effect was observed in MCF-7 cells with unloaded nanocarriers [TiO_2_-PAC_16_]@shell**1** and [Al_2_O_3_-PAC_16_]@shell**1**. However, the X-ray induced depolarization of the MMP in cancer cells with quercetin and 7-amino-4-methylcoumarin loaded nanocarriers was remarkably large. This confirms that the X-ray triggered release of the anticancer drugs. In MCF-10 A cells (Figure 8B) the MMP did not significantly change independently of incubation of the cells with or without nanocarriers. A decrease of the MMP could lead to the release of cytochrome c out of the mitochondria which might activate the apoptotic pathways [27]. The induction of apoptosis or other cell death pathways is associated with the fragmentation of the DNA. DNA fragments can be stained with the TUNEL assay. The X-ray treatment was found to increase the fraction of TUNEL stained MCF-7 and MCF-10 A cells in nanoparticle-free medium (Figure 8C,D and Figure S11). X-ray irradiated MCF-7 cells, when incubated with [TiO_2_-PAC_16_]@guest **G1**@shell**1**, [TiO_2_-PAC_16_]@guest **G2**@shell**1** and [Al_2_O_3_-PAC_16_]@guest **G1**@shell**1**, exhibit a drastic increase of TUNEL staining (Figure 8C). Quercetin and 7-amino-4-methylcoumarin did not only depolarize the MMP but also caused cell death, most probably apoptosis, via the mitochondrial pathway. In contrast, we did not observe any increase in DNA fragmentation in irradiated MCF-10 A cells containing drug loaded nanocarriers (Figure 8D). The antioxidant effect of quercetin and 7-amino-4-methylcoumarin apparently protect the non-tumorigenic MCF-10 A cells against X-ray induced destruction.

### 3.6. Survival Curves of the Cells with and without Nanocarriers

**Figure 9:**
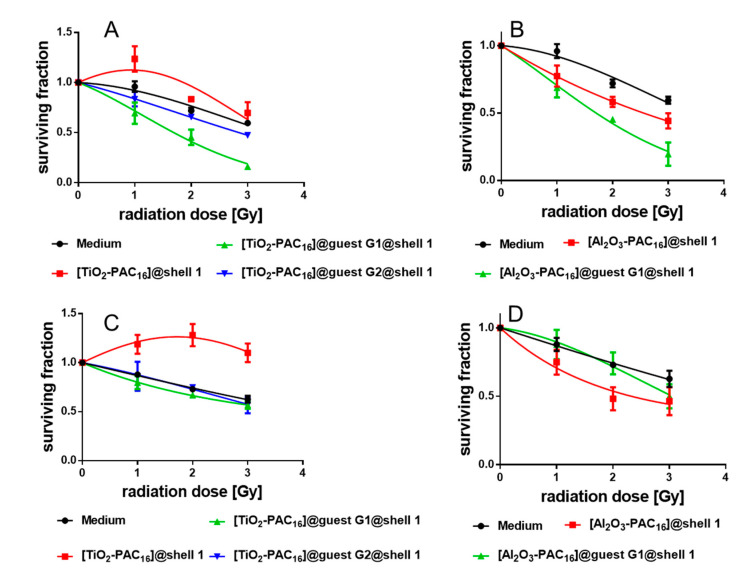
Cell survival curves of MCF-7 cells treated with unloaded and loaded TiO_2_ nanocarriers (**A**) and Al_2_O_3_ nanocarriers (**B**) and survival curves of the MCF-10 A cells treated with unloaded and loaded TiO_2_ nanocarriers (**C**) and Al_2_O_3_ nanocarriers (**D**).

To elucidate the radio-sensitizing potential of the various nanocarriers, the clonogenic cell survival assay was performed. The survival curves of the unloaded [TiO_2_-PAC_16_]@shell**1** (red line in Figure 9A,C) lies above the survival curve of the cells in medium without any nanocarriers (black line). The dose modifying factor (DMF) was calculated at a survival rate of 50%. All DMF values above 1 indicate radio-protection and below 1 radio-sensitization. The DMF value with 1.51 clearly identifies the [TiO_2_-PAC_16_]@shell**1** as radio-protector for the non-tumorigenic MCF-10 A cells. Unfortunately, the survival curve of [TiO_2_-PAC_16_]@shell**1** loaded cancer MCF-7 cells are also above the medium survival curve. With a DMF value of 0.99, the SbS coated TiO_2_ nanocarriers did not exhibit any radio-sensitizing effect, which is in contrast to other TiO_2_ nanostructures reported in literature [34,35,36,37]. However, the [TiO_2_-PAC_16_]@guest **G1**@shell**1** and [TiO_2_-PAC_16_]@guest **G2**@shell**1** internalized by the MCF-7 cells under X-ray exposure significantly reduced the survival of these cells (Figure 9A). With a DMF of 0.47, the nanocarriers loaded with quercetin acted as a more powerful radio-sensitizer than nanocarriers loaded with 7-amino-4-methylcoumarin (DMF 0.84). The survival curves of [Al_2_O_3_-PAC_16_]@shell**1** lie below the survival curves of the cells in medium (Figure 9B,D). Al_2_O_3_ NPs develop a cytotoxic and genotoxic effect [57,58,59] and may also increase mitochondria-mediated oxidative stress [60]. As already shown by the viability studies, the cell viability of MCF-7 cells and MCF-10 A cells with [Al_2_O_3_-PAC_16_]@shell**1** decreased after irradiation (Figure 6B,C). The rise in mitochondria-mediated oxidative stress was validated only in MCF-10 A cells treated with [Al_2_O_3_-PAC_16_]@shell**1**, in which X-radiation elevated the ROS formation (Figure 7C). However, Al_2_O_3_-PAC_16_]@shell**1**, when loaded with quercetin, decreased the survival rate of cancerous MCF-7 cells even more (Figure 9B). Interestingly, quercetin protected the non-tumorigenic MCF-10 A cells from the toxic effect of the Al_2_O_3_ nanocarriers, and the survival rate of MCF-10 A cells with [Al_2_O_3_-PAC_16_]@guest **G1**@shell**1** was boosted (Figure 9D). All DMF values are listed in the Supplementary Table S4. Both the TiO_2_- and Al_2_O_3_-based nanocarriers loaded with quercetin did not only perform as excellent radio-sensitizer for MCF-7 cells and did not damage the MCF-10 A cells. [TiO_2_-PAC_16_]@guest **G1**@shell**1** (DMF 0.47) is even a better radio-sensitizer than [Al_2_O_3_-PAC_16_]@guest **G1**@shell**1** (DMF 0.50) and simultaneously acted as radio-protector for MCF-10 A cells (DMF 1.21). In a nutshell, the [TiO_2_-PAC_16_]@guest **G1**@shell**1** are highly promising anticancer agents for combined cancer therapies.

## 4. Conclusions

Our research goal was to develop novel drug delivering nanoparticles for combined cancer chemo- and radiotherapy. We “functionalized” TiO_2_ and Al_2_O_3_ nanoparticles by encapsulating them in an amphiphilic bilayer shell that allow the incorporation of hydrophobic anticancer drugs. The amphiphilic bilayer shell was prepared by successfully elaborating a novel shell-by-shell technique. The nanoparticles were firstly covalently functionalized with hydrophobic hexadecylphosphonic acid (PAC_16_). A second shell composed of amphiphilic sodium benzenesulfonate was formed by self-aggregation which was driven by hydrophobic interactions between the dodecylbenzene moiety and the hydrophobic first shell. This resulting bilayer (SbS) provided hydrophobic pockets suited for the adsorption of hydrophobic drugs. The resulting SbS-coated TiO_2_ and Al_2_O_3_ NPs, performing as nanocarriers, were loaded with quercetin (**G1**), 7-amino-4-methylcoumarin (**G2**), and perylendiimide (**G3**). The incorporation of these hydrophobic drugs in hydrophobic pockets of the bilayer around the nanocarriers was confirmed by an increase in the hydrodynamic size of the loaded nanocarriers compared to the unloaded nanocarriers. In addition, the initially white nanocarrier dispersion became colored after being loaded with **G1**, **G2**, and **G3**.

Furthermore, we could prove that the nanocarriers, when incorporating quercetin or 7-amino-4-methylcoumarin, are biocompatible for the non-tumorigenic MCF-10 A cells as well as for the cancerous MCF-7 cells. These results confirm the stable incorporation of the hydrophobic drug in the bilayer. We could unambiguously demonstrate that an external stimulus is needed to release the drugs in the cytoplasm. Irradiation with a single dose of 1 Gy triggers the release of the encapsulated drugs. Quercetin and other flavonoids may act as both anti-oxidant and pro-oxidant, which depends on their intracellular concentration. MCF-10 A cells, uptaking a smaller concentration of quercetin-loaded nanocarriers than MCF-7 cells, were obviously protected against the impact of X-radiation. This indicates the radio-protecting effect of quercetin after being released into the cytoplasm. In contrast, the X-ray induced release of quercetin in MCF-7 cells led to enhanced ROS formation, which indicates their pro-oxidative effect in higher concentrations.

Furthermore, we could show that X-ray treatment of both kinds of cells MCF-7 and MCF-10 A depolarized the MMP. This MMP depolarization was found to be further increased by intracellular quercetin and 7-amino-4-methylcoumarin. In addition, the results from the TUNEL assay on cells that were exposed to X-rays and contained drug loaded nanocarriers confirmed that the drugs develop their anti-tumor effects.

The DMF values obtained for quercetin loaded nanocarriers corroborated the impressive radio-sensitizing potential of quercetin. All these results demonstrate, that quercetin-loaded, SbS-coated TiO_2_ nanocarriers represent highly efficient, multifunctional nanoagents for application in cancer radio- and chemotherapy.

## Figures and Tables

**Table 1:** Summary of the hydrodynamic diameter, zeta potential and loading capacity (LC) of the Shell-by-Shell (SbS) coated nanocarrier assemblies that are loaded with guest molecules **G1** (quercetin), **G2** (7-amino-4-methylcoumarin), and **G3** (perylendiimide).

Nanocarrier	Size in Water (nm)	Size in Medium (nm)	Zeta Potential (mV)	LC (%)
[TiO_2_-PAC_16_]@guest **G1**@shell**1**	106	197	−23.6 ± 2.2	7.1
[TiO_2_-PAC_16_]@guest **G2**@shell**1**	115	209	−38.5 ± 1.9	
[TiO_2_-PAC_16_]@guest **G3**@shell**1**	100	195	−22.3 ± 1.0	3.1
[Al_2_O_3_-PAC_16_]@guest **G1**@shell**1**	130	186	−35.0 ± 2.8	4.6
[Al_2_O_3_-PAC_16_]@guest **G3**@shell**1**	143	194	−35.0 ± 2.8	5.3

## Supplementary Materials

The following are available online at https://www.mdpi.com/2306-5354/7/4/126/s1. Figure S1: Synthetic pathway to the perylendiiimide (PDI) guest molecule **G3**, BET measurements of TiO_2_ and Al_2_O_3_ NPs [38,61], Figure S2: A + B. BET analysis of aluminum oxide NPs [21], Figure S3: Optical properties of [TiO_2_-PAC_16_]@shell**1** (1), [TiO_2_-PAC_16_]@ guest **G1** @shell**1** (2), [TiO_2_-PAC_16_]@ guest **G2** @shell**1** (3) and [TiO_2_-PAC_16_]@ guest **G3** @shell 1 (4) under day-light (A) and UV-light (B) conditions, Optical properties of [Al_2_O_3_-PAC_16_]@shell**1** (5), [Al_2_O_3_-PAC_16_]@ guest **G1** @shell**1** (6) and [Al_2_O_3_-PAC_16_]@ guest **G3** @shell**1** (7) under day-light (C) and UV-light (D) conditions, Figure S4: UV/Vis spectrum of the unloaded nanocarrier system [TiO_2_-PAC_16_]@shell**1**, loaded nanocarrier system [TiO_2_-PAC_16_]@guest **G1**@shell**1** and loaded nanocarrier system [TiO_2_-PAC_16_]@guest **G2**@shell**1**, Figure S5: Hydrodynamic diameter of the unloaded nanocarrier system [TiO_2_-PAC_16_]@shell**1** and loaded nanocarrier system [TiO_2_-PAC_16_]@ guest **G2** @shell**1**, Figure S6: Hydrodynamic diameter of the unloaded nanocarrier system [TiO_2_-PAC_16_]@shell**1** and loaded nanocarrier system [TiO_2_-PAC_16_]@ guest G1 @shell**1**, [TiO_2_-PAC_16_]@ guest **G2** @shell**1** and [TiO_2_-PAC_16_]@ guest **G3** @shell**1** in medium with 10% serum, Figure S7: Hydrodynamic diameter of the unloaded nanocarrier system [Al_2_O_3_-PAC_16_]@shell**1** and loaded nanocarrier system [Al_2_O_3_-PAC_16_]@ guest **G1** @shell**1** and [Al_2_O_3_-PAC_16_]@ guest **G3** @shell**1** in medium with 10% serum, Figure S8: MALDI experiment on loaded nanocarrier [TiO_2_-PAC_16_]@guest **G3** @shell**1**, Figure S9: Fluorescence microscope image of MCF-10 A cells with [Al_2_O_3_ PAC_16_]@guest **G3**@shell**1**, Figure S10: Biocompatibility of the hydrophobic drugs solved in DMSO without any nanocarriers for the MCF-7 and MCF-10A cells, Figure S11: Positive and negative control in the TUNEL assay, Table S1: BET analysis of titanium oxide NPs [19,23], Table S2: Hydrodynamic diameter and zeta potential values of the unloaded nanocarrier systems, Table S3: Polydispersity Index (PDI) of the various DLS measurements, Table S4: DMF values of the various nanocarrier systems.
