# Supplementary material for: Encapsulation of Hydrophobic Drugs in Shell-by-Shell Coated Nanoparticles for Radio- and Chemotherapy—An In Vitro Study
Source: Bioengineering (Basel). 2020 Oct 12;7(4):126. doi: 10.3390/bioengineering7040126 (PMC7712138; doi:10.3390/bioengineering7040126)
Supplement: Supplementary file 1 [file bioengineering-07-00126-s001.pdf]

Supplementary Materials

Encapsulation of Hydrophobic Drugs in Shell-by-Shell Coated Nanoparticles for Radio- and Chemotherapy—an In Vitro Study

Stefanie Klein [1,*], Tobias Luchs [2], Andreas Leng [2], Luitpold V. R. Distel [3], Winfried Neuhuber [4] and Andreas Hirsch [2]

[1] Department of Chemistry and Pharmacy, Physical Chemistry I and ICMM, Friedrich-Alexander University of Erlangen Nuremberg, Egerlandstr. 3, D-91058 Erlangen, Germany

[2] Department of Chemistry and Pharmacy, Chair of Organic Chemistry II, Friedrich-Alexander University of Erlangen Nuremberg, Nikolaus-Fiebiger-Str. 10, D-91058 Erlangen, Germany; tobias.luchs@fau.de (T.L.), andreas.leng@fau.de (A.L.), andreas.hirsch@fau.de (A.H.)

[3] Department of Radiation Oncology, Friedrich-Alexander University of Erlangen Nuremberg, Universitätsstr. 27, D-91054 Erlangen, Germany; luitpold.distel@uk-erlangen.de

[4] Institute of Anatomy and Cell Biology, Chair of Anatomy I, Friedrich-Alexander university of Erlangen Nuremberg, Krankenhausstr. 9, D-91054 Erlangen, Germany; winfried.neuhuber@fau.de

* Correspondence: stefanie.klein@fau.de; Tel.: +49-9131-8527508

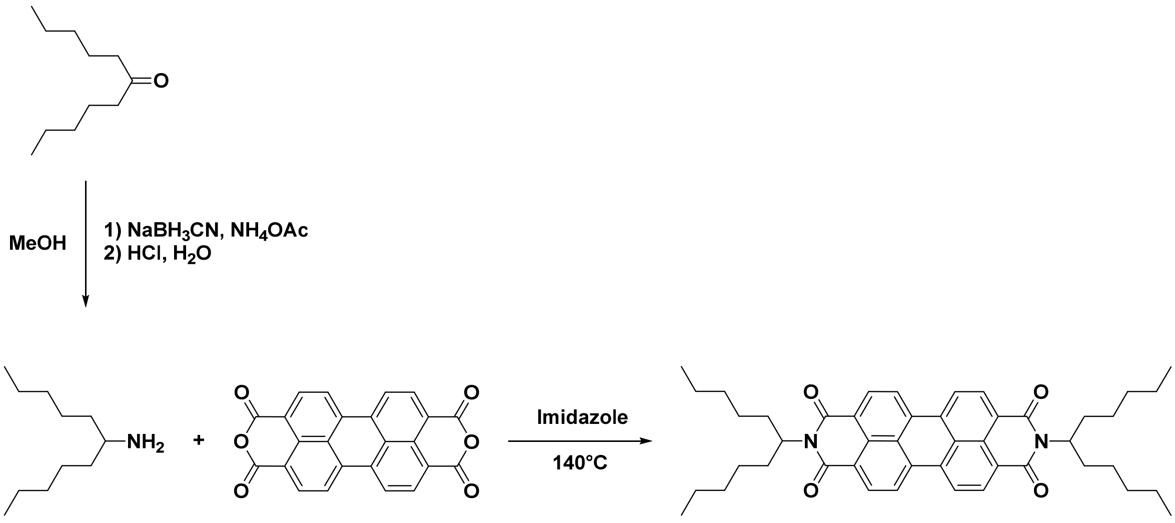

G3

Figure S1. Synthetic pathway to the perylenediimide (PDI) guest molecule **G3**.

1. -Pentylhexylamine [61]

6-Undecanone (5.03 g, 29.5 mmol) and NH_4OAc (20.1 g, 260 mmol, 8.8 eq.) were dissolved in methanol (150 mL) and stirred for 1.5 h. Subsequently, $NaCNBH_3$ (1.12 g, 17.8 mmol, 0.6 eq.) was added and the reaction was stirred for 3 d. HCl (conc., 30 mL) was added and the solvent was removed. The residue was dissolved in H_2O and KOH was added until pH > 10.5. The mixture was extracted with DCM (3 x 100 mL) and the combined organic layers were dried in vacuo. The crude product was purified by vacuum distillation (150 °C, 4.5 x 10^{-2} mbar) to obtain title compound as a colorless oil (3.17 g, 18.5 mmol, 62 %).

^{1}H-NMR ($CDCl_3$, 400 MHz, 25 °C): δ = 2.62 – 2.57 (m, 1H), 1.33 – 1.13 (m, 16H), 1.08 (s, 2H), 0.81 (t, J = 8 Hz, 6H) ppm.

^{13}**C-NMR** ($CDCl_3$, 101 MHz, 25 °C): δ = 51.4 (CH), 38.3, 32.2, 26.0, 22.8 (CH2), 14.2 (CH3) ppm.

G3 [38]

A flask was charged with perylene-3,4,9,10-tetracarboxylic dianhydride (2.88 g, 7.35 mmol, 1 eq.), 1-Pentylhexylamine (3.02 g, 17.6 mmol, 2.4 eq.) and imidazole under N_2-atmosphere and heated to 130 °C. After 1.5 h the reaction mixture was allowed to reach rt and H_2O (100 mL) was added. The aqueous layer was extracted with DCM (4 x 80 mL) and the combined organic layers were dried in vacuo. The obtained crude product was purified by plug chromatography to obtain title compound **G3** as a red solid (4.64g, 6.64 mmol, 90 %).

^{1}**H-NMR** ($CDCl_3$, 400 MHz, 25 °C): δ = 8.60 – 8.54 (m, 8H), 5.16 – 5.08 (m, 2H), 2.22 – 2.13 (m, 4H), 1.84 – 1.75 (m, 4H), 1.30 – 1.16 (m, 24H), 0.76 (t, J = 7.2 Hz, 12H) ppm.

^{13}**C-NMR** ($CDCl_3$, 101 MHz, 25 °C): δ = 164.56, 163.56 (C=O), 134.4, 131.8, 131.1, 129.5, 126.4, 122.9 (CAr), 54.8 (CH), 32.3, 31.8, 26.7, 22.6 (CH2), 14.0 (CH3) ppm.

BET Measurements:

The specific surface area of the utilized Titanium Oxide NPs was measured by BET analysis and was previously reported in publications of our group [19,23]:

Table S1. BET analysis of titanium oxide NPs

Relative Pressure	Volume @STP cc/g	Relative Pressure	Volume @STP cc/g
1.47970E-03	−2.45300E-01	8.66152E-01	5.54425E+01
1.47970E-03	−2.86100E-01	8.95600E-01	7.06593E+01
1.64672E-03	−3.06200E-01	9.26835E-01	1.11654E+02
4.12733E-03	5.10000E-01	9.62886E-01	1.94618E+02
4.92312E-03	6.67200E-01	9.91127E-01	2.04949E+02
6.96989E-03	9.98100E-01	9.93626E-01	2.06727E+02
9.61624E-03	1.29910E+00	9.95307E-01	2.14565E+02
1.22490E-02	1.55840E+00	9.51592E-01	1.99526E+02
3.51518E-02	2.91760E+00	9.50296E-01	1.99226E+02
6.87770E-02	4.22100E+00	9.22516E-01	1.87317E+02
1.03359E-01	5.38010E+00	8.85390E-01	9.06327E+01
1.41452E-01	6.59000E+00	8.49551E-01	5.81823E+01
1.73343E-01	7.58370E+00	8.00426E-01	4.13479E+01
2.05954E-01	8.58630E+00	7.73412E-01	3.65499E+01
2.40930E-01	9.63940E+00	7.39636E-01	3.21058E+01
2.74210E-01	1.06291E+01	6.98742E-01	2.84158E+01
3.04949E-01	1.15244E+01	6.53819E-01	2.53734E+01
3.38728E-01	1.25072E+01	6.17752E-01	2.33308E+01
3.74576E-01	1.35624E+01	5.77336E-01	2.13060E+01
4.07380E-01	1.45282E+01	5.40498E-01	1.97439E+01
4.50741E-01	1.59212E+01	5.02737E-01	1.82679E+01
4.81954E-01	1.69519E+01	4.68978E-01	1.70105E+01
5.13729E-01	1.80645E+01	4.27950E-01	1.55520E+01
5.47527E-01	1.93768E+01	3.90748E-01	1.42885E+01
5.80398E-01	2.07663E+01	3.58999E-01	1.32321E+01
6.22837E-01	2.28402E+01	3.22523E-01	1.20873E+01
6.52489E-01	2.44504E+01	2.83010E-01	1.08350E+01
6.90063E-01	2.68718E+01	2.73332E-01	1.05156E+01
7.19672E-01	2.93519E+01	2.51907E-01	9.86010E+00
7.56272E-01	3.29118E+01	2.11190E-01	8.51640E+00
7.86376E-01	3.67613E+01	1.83723E-01	7.58530E+00
8.40683E-01	4.72766E+01	1.57144E-01	6.69000E+00

MBET summary: Slope = 63.547, Intercept = 1.106e+01, Correlation coefficient, r = 0.999949, C constant = 6.748, Surface Area = 46.680 ~m^2/g.

The specific surface area of the utilized aluminum oxide NPs was measured by BET analysis and was previously reported in publications of our group [21]:

A

Quantachrome QuadraWin - Data Acquisition and Reduction
for QuadraSorb SI
©2000-11, Quantachrome Instruments. All rights reserved.
version 5.05

**Analysis**		**Report**	
**Operator:** Al	**Date:**6/21/2018	**Operator:** Al	**Date:**2018/06/22
**Sample ID:** AlOx	**Filename:**	QSf652.QPS	

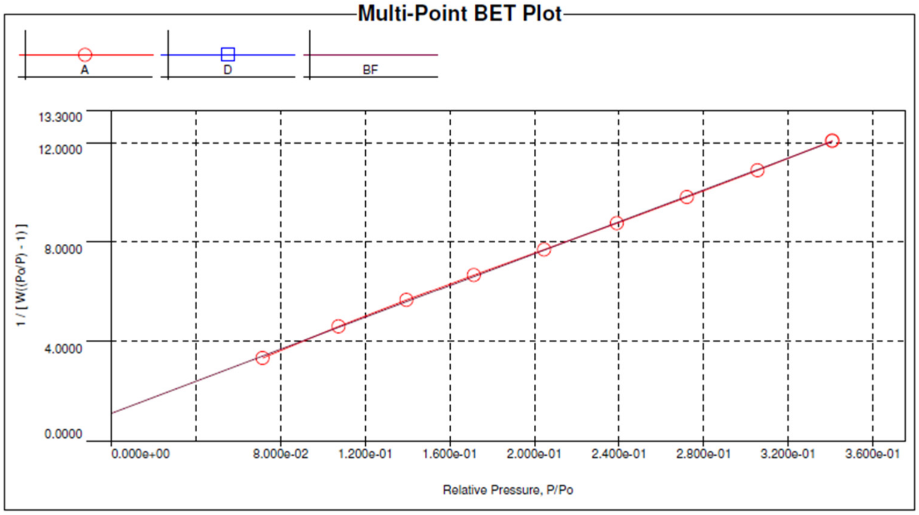

B

Quantachrome QuadraWin - Data Acquisition and Reduction
for QuadraSorb SI
©2000-11, Quantachrome Instruments. All rights reserved.
version 5.05

**Analysis**		**Report**	
**Operator:** Al	**Date:**6/21/2018	**Operator:** Al	**Date:**2018/06/22
**Sample ID:** AlOx	**Filename:**	QSf652.QPS	

Alpha-s summary

**Slope =**	60.107
**Alpha-S Intercept =**	-25.743 cc/g
**Correlation coefficient =**	0.998
**Micropore volume =**	-0.040 cc/g

Volume/Area summary

Surface Area Data

MultiPoint BET	1.049e+02 m²/g
BJH method cumulative adsorption surface area	9.270e+01 m²/g
BJH method cumulative desorption surface area	1.260e+02 m²/g
t-method external surface area	1.049e+02 m²/g
DR method micropore area	7.761e+01 m²/g
DFT cumulative surface area	1.088e+02 m²/g

Pore Volume Data

Total pore volume for pores with Diameter less than 364.61 nm at P/Po = 0.994723	7.365e-01 cc/g
BJH method cumulative adsorption pore volume	7.294e-01 cc/g
BJH method cumulative desorption pore volume	7.458e-01 cc/g
DR method micropore volume	2.758e-02 cc/g
DFT method cumulative pore volume	6.694e-01 cc/g

Pore Size Data

Average pore Diameter	2.808e+01 nm
BJH method adsorption pore Diameter (Mode Dv(d))	4.068e+01 nm
BJH method desorption pore Diameter (Mode Dv(d))	3.025e+01 nm
DR method micropore Pore width	1.693e+00 nm
DFT pore Diameter (Mode)	2.376e+01 nm

Figure S2. A + B. BET analysis of aluminum oxide NPs.

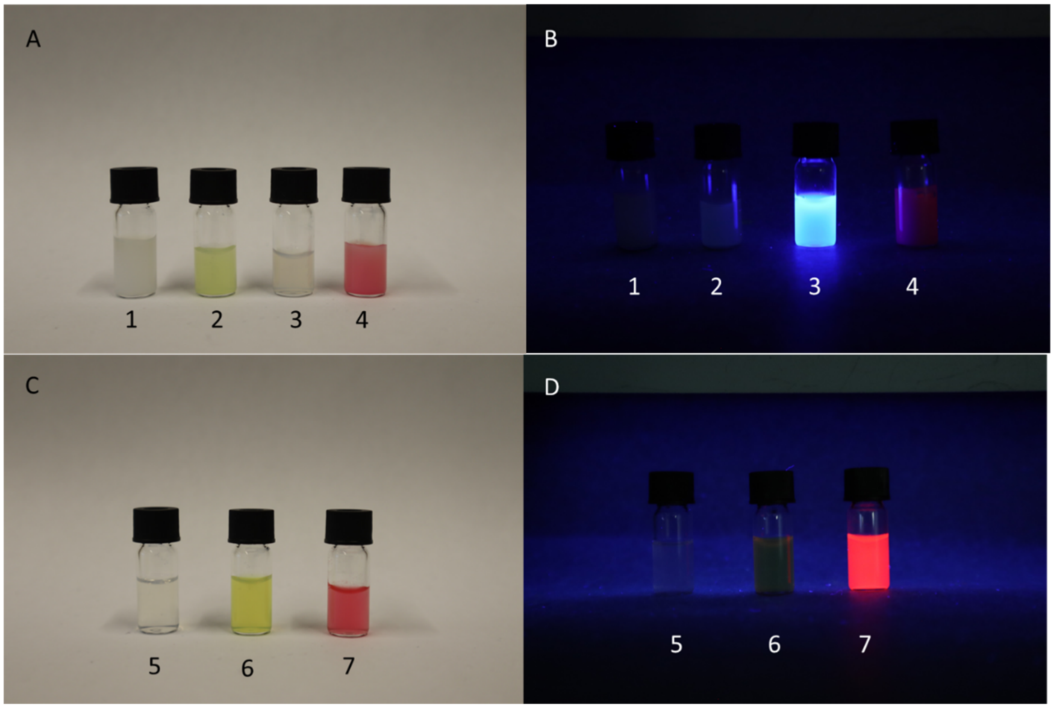

Figure S3. Optical properties of [TiO_2-PAC_{16}]@shell **1** (1), [TiO_2-PAC_{16}]@ guest **G1** @shell **1** (2), [TiO_2-PAC_{16}]@ guest **G2** @shell **1** (3) and [TiO_2-PAC_{16}]@ guest **G3** @shell **1** (4) under day-light (**A**) and UV-light (**B**) conditions, Optical properties of [Al_2O_3-PAC_{16}]@shell **1** (5), [Al_2O_3-PAC_{16}]@ guest **G1** @shell **1** (6) and [Al_2O_3-PAC_{16}]@ guest **G3** @shell **1** (7) under day-light (**C**) and UV-light (**D**) conditions.

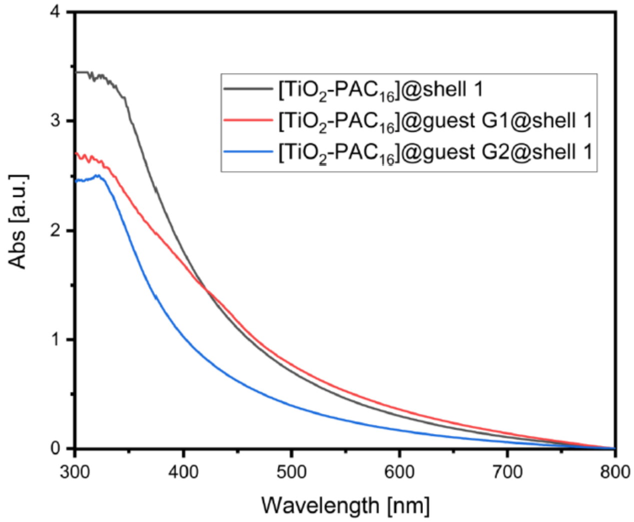

Figure S4. UV/VIS spectrum of the unloaded nanocarrier system [TiO_2-PAC_{16}]@shell **1**, loaded nanocarrier system [TiO_2-PAC_{16}]@guest **G1**@shell **1** and loaded nanocarrier system [TiO_2-PAC_{16}]@guest **G2**@shell **1**.

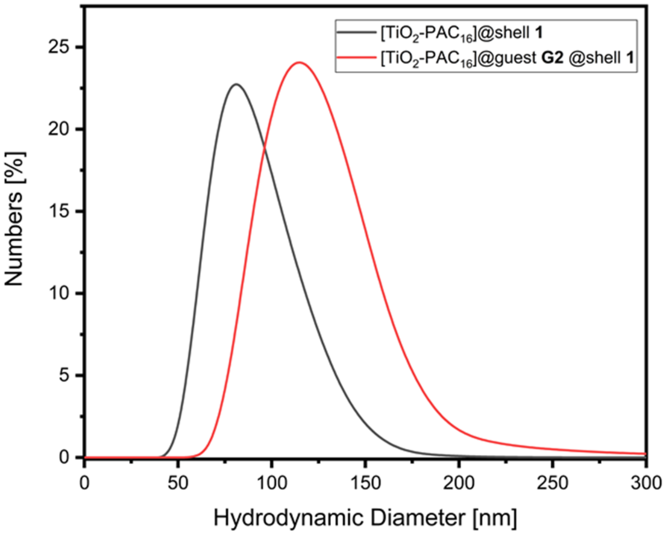

Figure S5. Hydrodynamic diameter of the unloaded nanocarrier system [TiO_2-PAC_{16}]@shell 1 and loaded nanocarrier system [TiO_2-PAC_{16}]@ guest **G2** @shell 1.

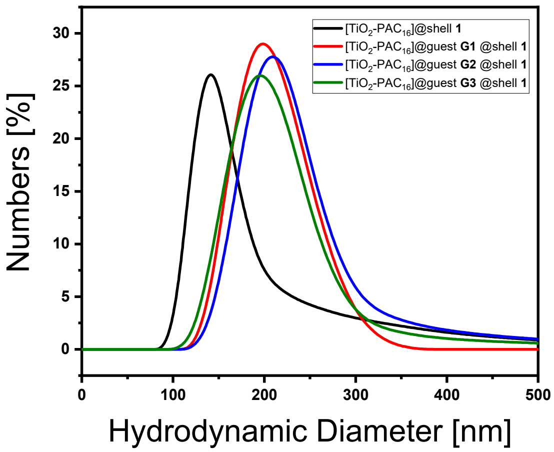

Figure S6. Hydrodynamic diameter of the unloaded nanocarrier system [TiO_2-PAC_{16}]@shell **1** and loaded nanocarrier system [TiO_2-PAC_{16}]@ guest **G1** @shell **1**, [TiO_2-PAC_{16}]@ guest **G2** @shell **1** and [TiO_2-PAC_{16}]@ guest **G3** @shell **1** in medium with 10 % serum.

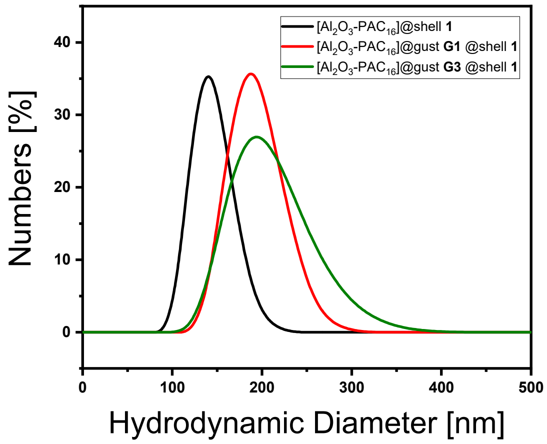

Figure S7. Hydrodynamic diameter of the unloaded nanocarrier system [Al_2O_3-PAC_{16}]@shell **1** and loaded nanocarrier system [Al_2O_3-PAC_{16}]@ guest **G1** @shell **1** and [Al_2O_3-PAC_{16}]@ guest **G3** @shell **1** in medium with 10 % serum.

Table S2. Hydrodynamic diameter and zeta potential values of the unloaded nanocarrier systems.

Nanocarrier System	Size in Water(nm))	Size in Medium (nm)	Zeta Potential (mV)
[TiO_2-PAC_{16}]@shell **1**	81	142	-39.1 ± 0.9
[Al_2O_3-PAC_{16}]@shell **1**	104	140	-31.8 ± 0.8

Table S3. Polydispersity Index (PDI) of the various DLS measurements.

Nanocarrier System	PDI in Water	PDI in Medium
[TiO_2-PAC_{16}]@shell **1**	0.161 ± 0.013	0.267 ± 0.010
[TiO_2-PAC_{16}]@ guest **G1**@shell **1**	0.341 ± 0.018	0.355 ± 0.032
[TiO_2-PAC_{16}]@ guest **G2**@shell **1**	0.137 ± 0.007	0.238 ± 0.036
[TiO_2-PAC_{16}]@ guest **G3**@shell **1**	0.217 ± 0.015	0.227 ± 0.013
[Al_2O_3-PAC_{16}]@shell **1**	0.198 ± 0.020	0.353 ± 0.046
[Al_2O_3-PAC_{16}]@ guest **G1**@shell **1**	0.243 ± 0.004	0.233 ± 0.005
[Al_2O_3-PAC_{16}]@ guest **G2**@shell **1**	0.344 ± 0.022	0.617 ± 0.022

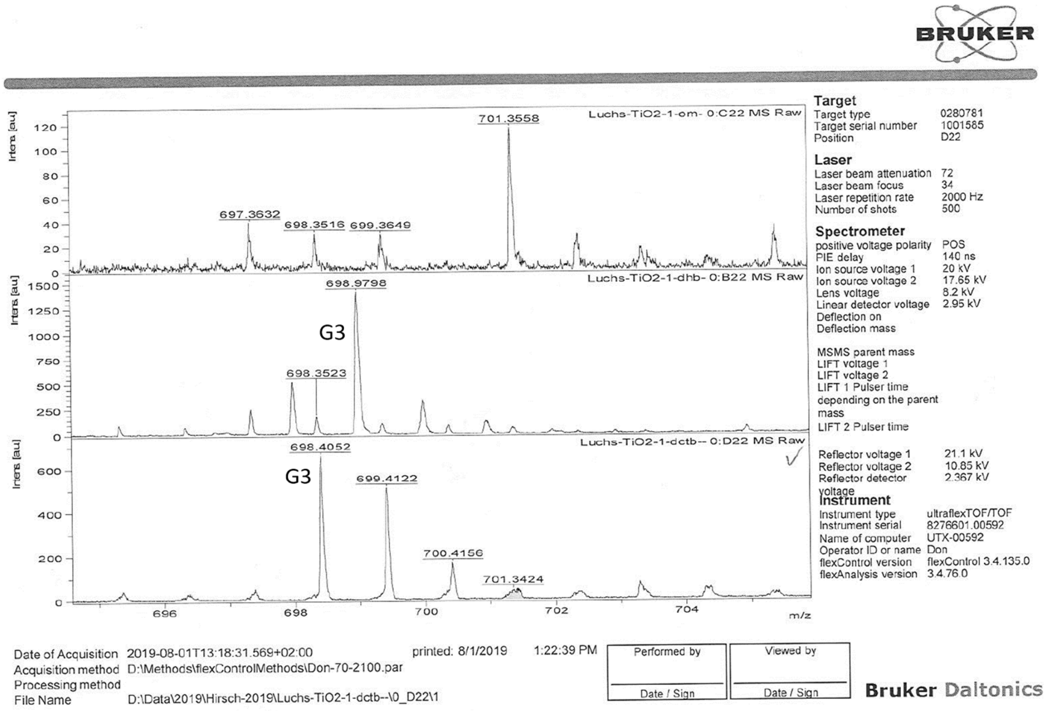

Figure S8. MALDI experiment on loaded nanocarrier [TiO_2-PAC_{16}]@guest **G3** @shell 1.

For further analytical verification of the guest incorporation a MALDI TOF experiment was conducted. An aqueous 0.15 wt % dispersion of [TiO_2-PAC_{16}]@guest **G3** @shell 1 was deposited on a sample plate, either without Matrix, together with dhb (dihydroxybenzoic acid) or together with dctb (trans-2-[3-(4-t-butyl-phenyl)-2-methyl-2-propenylidene]malononitrile). After evaporation of the solvent, the sample was introduced into the mass spectrometer and submitted to ionization. The samples without Matrix and with dhb as matrix displayed only traces of the guest molecule **G3**, while the sample with dctb as Matrix showed a clear signal with an m/z ratio of 698.4 matching the expected signal for **G3**. Therefore, this mass spectrometric experiment clearly verified the incorporation of hydrophobic guest molecules into the scaffold of SbS coated nanoparticles.

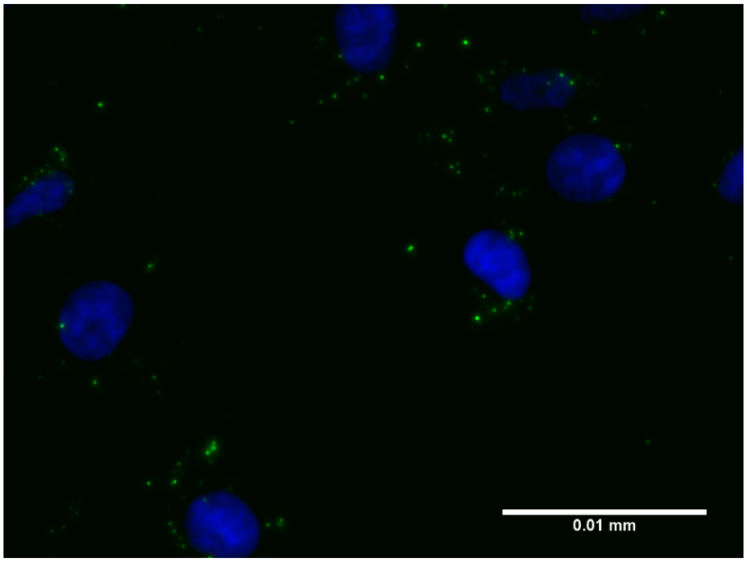

Figure S9. Fluorescence microscope image of MCF-10 A cells with [Al_2O_3-PAC_{16}]@guest **G3**@shell **1.**

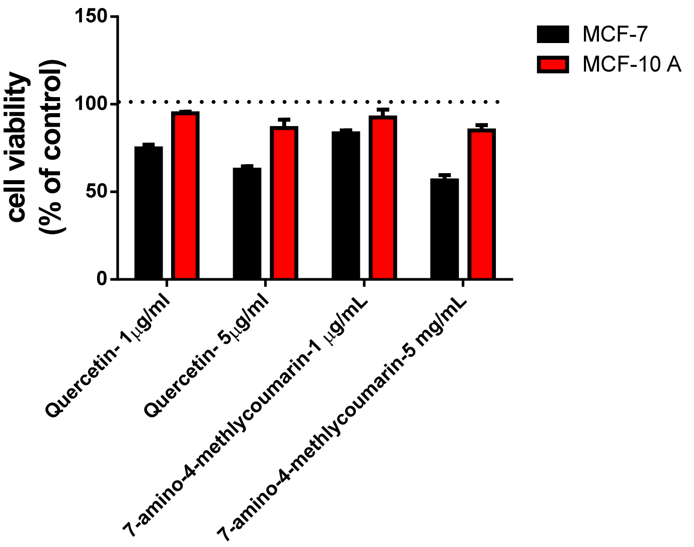

Figure S10. Biocompatibility of the hydrophobic drugs solved in DMSO without any nanocarriers for the MCF-7 and MCF-10A cells.

Table S4. DMF values of the various nanocarrier systems.

DMF Values	MCF-7	MCF-10A
[TiO_2-PAC_{16}]@shell 1	0.99	1.51
[TiO_2-PAC_{16}]@ guest **G1** @shell 1	0.47	1.21
[TiO_2-PAC_{16}]@ guest **G2** @shell 1	0.84	1.03
[Al_2O_3-PAC_{16}]@shell 1	0.75	0.69
[Al_2O_3-PAC_{16}]@ guest **G1** @shell 1	0.50	0.90

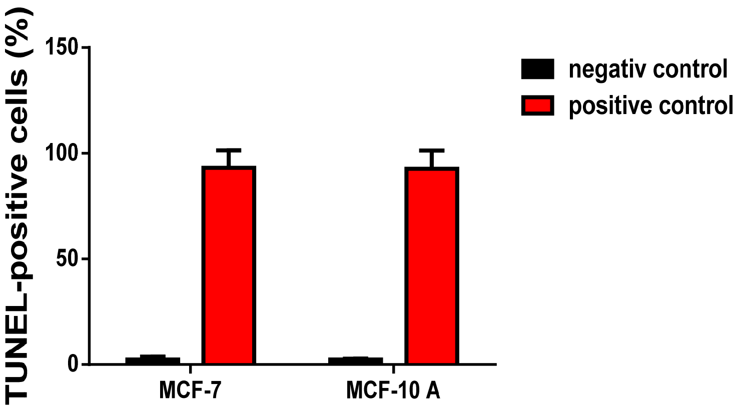

Figure S11. Positive and negative control in the TUNEL assay.
